# Malignant gastrointestinal stromal tumor presenting with hemoperitoneum in puerperium: report of a case with review of the literature

**DOI:** 10.1186/1477-7819-8-95

**Published:** 2010-11-07

**Authors:** Michail Varras, Nikolaos Vlachakos, Christodoulos Akrivis, Thivi Vasilakaki, Evangelia Skafida

**Affiliations:** 1 Department of Obstetrics and Gynecology, 'Tzaneio' General State Hospital, Piraeus, Greece; 2 Department of General Surgery, Tzaneio General State Hospital, Piraeus, Greece; 3 Department of Obstetrics and Gynecology, 'G. Chatzikosta' General State Hospital, Ioannina, Greece; 4 Department of Pathology, 'Tzaneio' General State Hospital, Piraeus, Greece

## Abstract

**Background:**

Gastrointestinal stromal tumors (GISTs) are mesenchymal tumors that develop in the wall of the gastrointestinal tract and their diagnosis during pregnancy or puerperium is extremely rare.

**Case:**

A 28-year old patient presented with acute abdomen due to hemoperitoneum from a large mass arising of the small intestine with distended vessels on its top and a ruptured superficial vessel bleeding into the peritoneal cavity. The patient was at the tenth postpartum day of her first pregnancy. The preoperative diagnosis was a possible ovarian or uterine mass. After an emergency exploratory laparotomy a segmental bowel resection was performed, removing the tumor with a part of 3-cm of the small intestine. Histology revealed GIST with maximum diameter of 13 cm and mitotic rates more than 5 mitoses per 50 high power fields with some atypical forms, indicating a high risk malignancy. Immunohistochemical staining of the tumor tissue demonstrated strongly positive reactivity to CD 117 (c-kit) and CD34 in almost all the tumor cells. The patient was treated with oral imatinib mesylate (Gleevec) 400 mg daily for one year. Three years after surgery, the patient was alive without evidence of metastases or local recurrence.

**Conclusion:**

Considering that only few patients with gastrointestinal stromal tumors have been reported in the obstetrical and gynecological literature, the awareness of such an entity by the obstetricians-gynecologists is necessary in order to facilitate coordinated approach with the general surgeons and oncologists for the optimal care of the patients.

## Introduction

Gastrointestinal stromal tumors (GISTs) are uncommon tumors that develop in the wall of the gastrointestinal tract and usually present in the fifth to seventh decade of life [1,2]. They account for approximately 0.1% to 3% of all gastrointestinal neoplasms, with an incidence of 1-20 per million and up to 30% of these are considered malignant [1,3,4]. The term gastrointestinal sromal tumor, first used by Mazur and Clark in 1983, encompasses a heterogeneous group of nonepithelial neoplasms composed of spindle or epithelioid cells, which display a range of differentiation [5]. Given the age distribution of occurrence, a diagnosis of gastrointestinal stromal tumor during pregnancy [6-8] or puerperium is very uncommon.

We hereby describe our experience of the case of a GIST discovered during the puerperium, in a 28-year old patient presented with acute abdomen due to spontaneous rupture of a superficial tumor vessel, an extremely rare complication and review the current literature.

## Case Report

**Figure 1:**
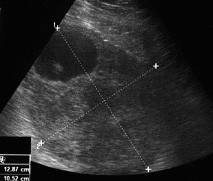
**Abdominal ultrasonography revealed a large mass measuring 12.85 × 10.52-cm with mixed echogenicity, occupying the pelvis and extending above the pubic symphysis; two cystic areas within the mass measuring approximately 4.30 × 4.97-cm and 3.54 × 3.66-cm are noted**.

**Figure 2:**
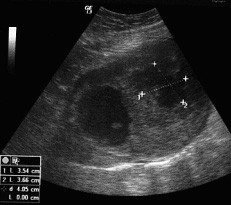
**Abdominal ultrasonography revealed a large mass measuring 12.85 × 10.52-cm with mixed echogenicity, occupying the pelvis and extending above the pubic symphysis; two cystic areas within the mass measuring approximately 4.30 × 4.97-cm and 3.54 × 3.66-cm are noted**.

**Figure 3:**
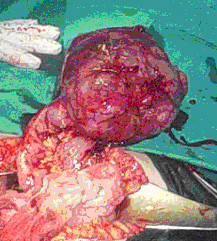
**Exploration of the peritoneal cavity revealed a large mass arising from the small intestine and growing exophytically out into the peritoneal cavity**. On the top of the mass, distended vessels were observed and a ruptured superficial vessel was actively bleeding into the abdominal cavity.

**Figure 4:**
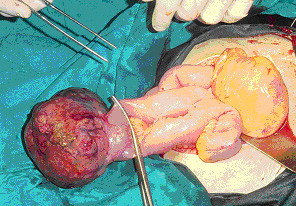
**Demonstration of the resection of segmental bowel; the tumor was removed with a part of 3-cm of the small intestine**.

A 28-year-old woman was brought to the emergency department of our hospital with severe lower abdominal pain, which became generalized and intolerable. The patient was at the tenth postpartum day of her first pregnancy and had no remarkable medical or surgical history. Also, the patient had no history of an irregular menstruation cycle. As the patient mentioned, during her pregnancy the uterus was considered too large for her gestational age and on routine ultrasounds a subserosal fibroid was suspected. The crown rump length was in accordance with her last menstrual period and the fetal growth was within the normal limits as well, according to the patient's information. She had a normal delivery at term at a Private Maternity Hospital of Athens. The patient denied any medical history of gastrointestinal symptoms such as emesis, melaena, abdominal pain or ileus during her pregnancy. At presentation, she was nauseous and had vomited a number of times. Physical examination revealed a pale, moderately obese young woman with a heart rate of 104 beats per minute, blood pressure 130/70 mmHg and temperature 36°C. Her abdomen was extensively distended, markedly tender with moderate spasm and rebound tenderness in both iliac fossae. Peristaltic sounds were diminished. Her blood count demonstrated a haemoglobin concentration of 9.4 g/dl, haematocrit 31%, white blood count 18,000 cells/ml with 89.7% polymorphonuclears and platelets 352,000/μl. Clotting time, bleeding time, serum liver enzymes and kidney function tests were within normal limits. L.D.H. was 297 U/l (normal rates 100-240 U/l). An abdominal ultrasound examination revealed a large mass measuring 12.85 × 10.52-cm with mixed echogenicity, occupying all the pelvis and extending above the pubic symphysis and from the midline to the left (Figures 1-2). Two cystic areas within the mass measuring approximately 4.30 × 4.97-cm and 3.54 × 3.66-cm were found (Figures 2). The ovaries were not visualized. Free fluid was present at the Morisson's space and the cul-de-sac with low levels of echogenic debris. Chest X-ray examination was negative. Under the diagnosis of hemoperitoneum from a possible ovarian or uterine mass, an immediate exploratory laparotomy was performed. At laparotomy with a vertical, midline infra-umbilical incision bloodstained fluid and blood clots in the peritoneal cavity were found. Further exploration revealed a large mass arising from the small intestine and growing exophytically out into the peritoneal cavity (Figure 3). On the top of the mass, distended vessels were observed and a ruptured superficial vessel was actively bleeding into the abdominal cavity; no other bleeding was indentified. Free fluid was obtained for cytology. One liter of fluid and blood clots were evacuated. A segmental bowel resection was performed, removing the tumor with a part of 3-cm of the small intestine (Figure 4). The abdominal cavity was irrigated and carefully inspected; the omentum, the ovaries and the uterus had normal macroscopic appearance and no visible findings suspicious of malignancy were found. The patient made a good recovery post-operatively.

**Figure 5:**
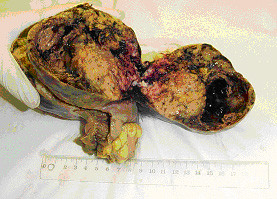
**The surgical specimen of the small intestine was a well-circumscribed tumor measuring 13 × 10 × 9-cm in size and located 3-cm from the nearest surgical margin**. The cut surface demonstrated whitish-gray solid parenchyma, with two cystic areas of degeneration with hemorrhagic fluid; the solid portion was soft in composition.

**Figure 6:**
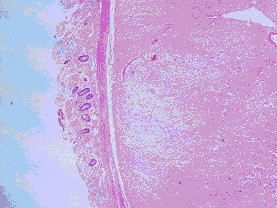
**Microscopically, the neoplasticcells were mainly spindle-shaped or partly epithelioid. Figures 13: H&E × 40; Figure 14: H&E × 100**.

**Figure 7:**
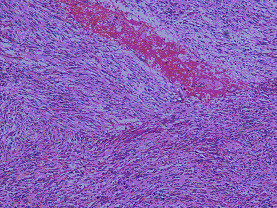
**Microscopically, the neoplasticcells were mainly spindle-shaped or partly epithelioid**. Figures 13: H&E × 40; Figure 14: H&E × 100.

**Figure 8:**
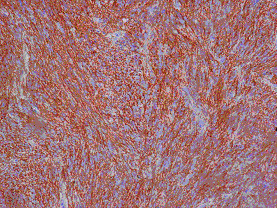
**Immunohistochemical staining of the tumor tissue demonstrated strongly positive reactivity to CD 117 (c-kit) in almost all the tumor cells**.

**Figure 9:**
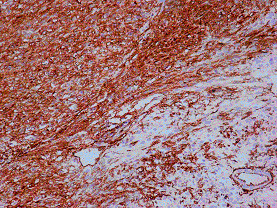
**Immunohistochemical staining of the tumor tissue demonstrated strongly positive reactivity to CD34 in almost all the tumor cells**.

Grossly, the surgical specimen of the small intestine showed a well-circumscribed tumor measuring 13 × 10 × 9-cm in size and located 3-cm from the nearest surgical martin (Figure 5). The external surface of the tumor showed pronounced appearance of its vessels. Part of the tumor was covered by the instinal musosa. The cut surface demonstrated whitish-gray solid parenchyma, with two cystic areas of degeneration with hemorrhagic fluid; the largest cyst measured 5-cm in its maximum diameter. The solid portion was soft in composition. Microscopically, the neoplastic cells were mainly spindle-shaped or partly epithelioid (Figures 6, 7). The mitotic rate was more than 5 mitoses per 50 HPFs (high power fields) with some atypical forms. The neoplastic stroma showed an important vascular component. There were some areas with necrosis, hemorrhage, and cystic degeneration. Immunohistochemical staining of the tumor tissue demonstrated strongly positive reactivity to CD 117 (c-kit) (Figure 8) and CD34 (Figure 9) in almost all the tumor cells, whereas a small percentage of the neoplastic cells was positive for α-smoth muscle actin. The immunostaining was negative for desmin, S-100 protein, and cytokeratins of high and low molecular weight. Cell proliferation by Ki-67 immunostaining was low. The tumor was diagnosed as a primary malignant gastrointestinal stromal tumor with high risk. The surgical margins of the intestinal specimen were negative for tumor cells. Cytologic examination of the peritoneal fluid obtained intraoperatively was negative for malignancy.

The patient's postoperative course was uneventful and she was treated with oral imatinib mesylate (Gleevec) 400 mg daily for one year. Three years after surgery for the primary gastrointestinal stromal tumor, the patient is alive. A recent CT scan of the upper and lower abdomen was negative for local recurrences of the disease or secondary metastases.

## Discussion

GISTs occur anywhere in the intestine, with the most common site being the stomach (50-60%), followed by the small intestine (20-30%), large bowel (10%), the oesophagus (5%), and only 5% elsewhere in the abdominal cavity such as in the mesentery, omentum or retroperitoneum [2,9].

GISTs may be detected during a gastroscopy as submucosal tumors or occasionally as incidental radiologic findings. The symptomatic GISTs of the esophagus typically present with dysphagia. Gastric and small intestinal GISTs often present with vague symptoms, but sometimes they cause upper gastrointestinal bleeding. Colorectal GISTs may manifest with lower gastrointestinal bleeding, colonic perforation, pain, obstruction or combination [4,10,11]. Also, fever or liver metastasis have described as first symptoms [12]. Rarely, they can present with intraperitoneal bleeding secondary to surface tumor ulteration [9,10]. Our patient presented clinically with pelvic pain and a pelvic mass diagnosed ultasonographically showing adequate fluid in the abdominal and pelvic cavities. The preoperative diagnosis was consistent with acute abdomen from a possible ovarian or uterine tumor. The ultrasonographic findings of GISTs are non-characteristic and therefore a preoperative presumptive diagnosis based on imaging is virtually impossible [9]. In our patient, during the operation, on the top of the mass a ruptured superficial vessel was found to bleed actively into the abdominal cavity and no other bleeding was indentified. It seems that the hemoperitoneum resulting from a solid mass is secondary to passive blood congestion, subsequent rupture of a superficial tumoral vessel and spontaneous internal bleeding [13]. The postpartum hyperfibrinolysis might play a role for the bleeding of the mass. A diagnosis of GIST during pregnancy is very uncommon [6-8]. Those few cases reported were symptomatic and found in the second half of the pregnancy, leading to an emergency cesarean section in one case due to fetal distress during laparotomy [6,7]. The delay in diagnosis of GISTs during pregnancy is normally due to the clinicians' reluctance to request diagnostic examinations during pregnancy and to the non-specific symptoms of the disease [8]. Our patient had a normal delivery at term at a Private Maternity Hospital of Athens, because no obstruction of labor had occurred obviously. Possibly, the large mass arising from the small intestine and growing exophytically out into the peritoneal cavity was removed outside the pelvis by the pregnant uterus. For the same reason the patient might have tolerated the high pressure during labor and the tumor did not stared bleeding during labor.

Macroscopically, GISTs are usually grey-white in appearance. They arise in the muscularis propria, and can grow either exophytically out into the peritoneum, or endophytically into the lumen of the gut [2]. Microscopically, GISTs are well-circumscribed smooth lobulated, uncapsulated tumours. They are composed of spindle cells or epithelioid cells, or a mixture of both, and may show areas of cystic degeneration, necrosis or focal hemorrhage [Sanjay et al 2004]. The hypothesis that GISTs originate from the primitive stem cell that can differentiate toward the interstitial cells of Cajal, has been advocated. Interstitial cells of Cajal are autonomous nerve-related GI pacemaker cells that regulate intestinal motility and are immunoreactive for a specific immunostain (CD117) of a c-kit proto-oncogene protein (KIT), which encodes for a transmembrane tyrosine-kinase receptor [14-18]. Mutations of c-kit proto-oncogen seem to produce overexpression of the KIT protein, which is responsible for the pathogenesis of GISTs [2]. Also, GISTs are often positive for CD34 and variably positive for smooth muscle actin [19]. Mesenchymal tumors of the uterus and ovaries are thought to rarely express c-kit, but if they do, the staining is usually focal, with fewer than 5% of cells been positive [9,20,12]. The final diagnosis of our patient was established based on the intestinal origin of the neoplasm and its histopathologic and immunohistochemical findings. The immunohistochemical analysis showed positive reactivity to the c-kit gene, CD34, a-smooth muscle actin, but no reactivity to S-100 protein.

Management depends on complete surgical resection, incomplete resection being associated with a median survival of less than 20 months. The wide margins of resection are not necessary as there is minimal local invasion and similarly lymphadenectomy is not routinely necessary as local lymph nodes are not usually affected [2]. Various systemic chemotherapeutic regimes, radiation and intraperitoneal chemotherapy has been used with little success [9]. However, Joensuu et al reported the first use of STI-571 (imatinib mesylate, Gleevec) in a case of recurrent metastatic GIST that failed extensive surgical therapy and chemotherapy [21]. The dramatic response in their case was documented both clinically and histologically. They also documented a marked decrease in tumor activity by 18FDG-PET scanning. Shortly after reporting the case, confirmatory data were published [22,23].

Prognostic factors indicating the possible malignant potential of gastrointestinal tumors are mitotic activity (>5 mitotic figures per 50 × high power field) and tumor size (>5-cm). Tumors that have the c-kit exon 11 mutation are also at greater risk [2]. Tumor rupture before or during surgery has also been linked to poor prognosis [9,4,24]. Factors as mucosal invasion and tumor necrosis have found to be related to increased risk of aggressive behavior, but their clinical value remains uncertain [24]. The spread pattern of gastrointestinal tumors with malignant potential shows predilection for liver metastasis and peritoneal dissemination. Therefore, the presence of hepatic or peritoneal lesions on presentation usually represents a sign of poor prognosis [4,9]. In addition, incomplete surgical resection is associated with a reduced survival [2]. Chemotherapy with imatinib (Gleevec, Novartis, Switzerland) was perfomed in our patient due to the large tumor size (>5-cm), and mitotic activity (>5/50 HPF). Reccurrence or metastasis after complete surgical resection may occur in more than two thirds of all gastrointestinal stromal tumors. Recurrence is usually local or peritoneal and often associated with liver metastases. Most recurrences occur within 2 years of the original tumor, although intervals of up to 10 years have been reported (2). In our case, three years after surgery, the patient was alive and the recent CT scan of the upper and lower abdomen showed no local recurrences of the disease or secondary metastases. Because of limited experience of GISTs diagnosed during pregnancy, no reference is made to the possibility of metastatic disease in the fetus or the developing of GIST *in utero *[6].

## Conclusions

Since only few patients with gastrointestinal stromal tumors have been reported in the obstetrical and gynecological literature [6,12,8,25-31], the awareness of such an entity by the obstetricians-gynecologists is necessary in order to include this in the differential diagnosis of minor gastrointestinal discomfort during pregnancy and in addition to facilitate coordinated approach with the general surgeons and oncologists for the optimal care of the patients. Complete surgical resection and immediate therapy with imatinib are associated with better survival of patients with such refractory tumors for radiotherapy and conventional chemotherapy. For GISTs diagnosed during pregnancy no reference is made to the possibility of metastatic disease in the fetus or the developing of GIST *in utero *[6]. For pregnant patients with suspicious tumor findings on ultrasonography, the ultrasound examination should determine the origin of the mass and its location, size and internal structure. For ovarian masses, color Doppler imaging should also be performed. Pelvic MRI with gadolinium injection can be performed after the first trimester to remove any doubt or to provide additional information if the ultrasound examination is not sufficient or as a tool for the assessment of cancer. Pelvic CT scanning is not indicated during pregnancy. Surgery should be immediate considered in cases of acute symptoms or suspicious tumors for malignancy [32]. The best predictor of whether a uterine leiomyoma cause problem during pregnancy is its location. Submucosal leiomyomas interfere with the implantation of the placenta, subserosal feiomyomas present with infarction, while leiomyomas in the cervix or at lower uterine segment may cause obstruction of the labor. Rarely, large submucosal nonpedunculated uterine leiomyoma can interfere with puerpurium by obstructing the passage of lochia and leading to haematometra and uterine atony [33].

## Consent

Written informed consent was obtained from the patient for publication of this case report and accompanying images. A copy of the written consent is available for review by the Editor-in-Chief of this journal.

## Competing interests

The authors declare that they have no competing interests.

## Authors' contributions

MV was the principal investigator and responsible for the original conception and design, has taken part in the operation, edited the manuscript, supervised the whole attempt and was responsible as well for images, correction, revision, and approval of the final version. NV has operated, was clinically responsible for patient's care and edited the manuscript. ChA has edited the manuscript. ThV was responsible for the histology consulting and pathology examination and has edited the manuscript. ES has diagnosed and edited the manuscript. All authors read and approved the final manuscript.
